# A review of accelerometer-derived physical activity in the idiopathic inflammatory myopathies

**DOI:** 10.1186/s41927-019-0088-1

**Published:** 2019-10-21

**Authors:** Alexander Oldroyd, Max A. Little, William Dixon, Hector Chinoy

**Affiliations:** 1 Centre for Epidemiology Versus Arthritis, The University of Manchester, Stopford Building, Oxford Road, Manchester, M13 9PT UK; 2 NIHR Manchester Biomedical Research Centre, Manchester Academic Health Science Centre, Manchester University NHS Foundation Trust, Manchester, UK; 3 Centre for Musculoskeletal Research, Manchester Academic Health Science Centre, University of Manchester, Manchester, UK; 4 Department of Rheumatology, Salford Royal NHS Foundation Trust, Salford, UK; 5 School of Computer Science, University of Birmingham, Birmingham, UK; 6 MIT Media Lab, Massachusetts Institute of Technology, Cambridge, MA USA

**Keywords:** Myositis, Muscle, Outcome measures, Human activities, Review, Accelerometry

## Abstract

**Background:**

The idiopathic inflammatory myopathies (IIMs) are a group of rare conditions characterised by muscle inflammation (myositis). Accurate disease activity assessment is vital in both clinical and research settings, however, current available methods lack ability to quantify associated variation of physical activity, an important consequence of myositis.

This study aims to review studies that have collected accelerometer-derived physical activity data in IIM populations, and to investigate if these studies identified associations between physical and myositis disease activity.

**Methods:**

A narrative review was conducted to identify original articles that have collected accelerometer-derived physical activity data in IIM populations. The following databases were searched from February 2000 until February 2019: Medline via PubMed, Embase via OVID and Scopus.

**Results:**

Of the 297 publications screened, eight studies describing accelerometer use in 181 IIM cases were identified. Seven out of the eight studies investigated juvenile dermatomyositis (JDM) populations and only one reported on an adult-onset population. Population sizes, disease duration, accelerometer devices used, body placement sites, and study duration varied between each study.

Accelerometer-derived physical activity levels were reduced in IIM cohorts, compared to healthy controls, and studies reported improvement of physical activity levels following exercise programme interventions, thus demonstrating efficacy.

Higher levels of accelerometer-derived physical activity measurements were associated with shorter JDM disease duration, current glucocorticoid use and lower serum creatine kinase. However, no clear association between muscle strength and accelerometer-derived physical activity measures was identified.

**Conclusions:**

The use of accelerometer-derived physical activity in IIM research is in its infancy. Whilst knowledge is currently limited to small studies, the opportunities are promising and future research in this area has the potential to improve disease activity assessment for clinical and research applications.

## Background

The idiopathic inflammatory myopathies (IIMs) are a group of rare (annual incidence of 1.5–10 per million person-years [1], prevalence of 14 per 100,000 [2]) autoimmune conditions that can cause widespread inflammation and damage [3, 4]. A number of IIM subtypes are recognised, including dermatomyositis (DM), juvenile DM (JDM), polymyositis (PM) and inclusion body myositis. The most common manifestation of the IIMs is muscle inflammation, termed “myositis”. Each episode of myositis, if left untreated, results in irreversible muscle breakdown, disability and early mortality [5, 6]. Therefore, in clinical settings, the ability to identify and quantify the severity of active myositis is imperative, to allow appropriate treatment with the aim of preventing damage. Further, the availability of valid measurements of myositis disease activity is essential in research settings, e.g. to evaluate the efficacy of interventions.

A number of measurements of myositis disease activity currently exist and include manual muscle testing via the MMT-8, serum creatine kinase (CK) levels and validated questionnaires, such as the Health Assessment Questionnaire Disability Index (HAQ-DI). JDM-specific disease activity can also be assessed by measures such as the Childhood Myositis Assessment Scale (CMAS), Childhood Health Assessment Questionnaire (CHAQ) and the Paediatric Quality of Life Inventory (PEDS-QL). A number of valid measurements of myositis disease activity have been assimilated into the International Myositis Assessment and Clinical Studies Group (IMACS) “Disease Activity Core Set Measures” [7], which is currently used as the gold-standard of myositis disease activity assessment in both clinical and research settings.

These measurements of myositis disease activity, although accurate, only capture specific aspects of disease activity, and do not necessarily objectively assess the patient-experienced consequence of myositis – namely reduced ability to carry out physical activities due to active muscle disease or irreversible muscle damage [8]. A qualitative study by Alemo Munters et al. identified that ability to carry out physical activities, including walking, participating in social activities and cycling were particularly affected in a myositis population [9]. Importantly, this study also identified that limitations of these physical activities are not wholly assessed in the HAQ-DI and Myositis Activities Profile (MAP) [10], two leading methods of patient-reported disease activity assessment - only 21% of reported disabilities were covered by the HAQ-DI and only 32% were covered by the MAP.

Objective assessment of physical activity may provide a novel method for myositis disease activity assessment. Here, we take the World Health organisation definition of physical activity as “any bodily movement produced by skeletal muscles that requires energy expenditure” [11]. Worsening myositis leads to reduced force generation capability predominantly of proximal limb muscles [12, 13]. Subsequent slower walking speed and reduced stride length, as reported by Siegel et al. [14], result in patient-reported walking difficulty, particularly whilst climbing stairs. A number of studies have confirmed the impact of myositis upon physical activity, along with the association between myositis disease activity and physical activity [15–17]. Alexanderson et al. showed that in a myositis cohort, within the first year after diagnosis and treatment initiation, improvement of the Functional Index of myositis test, a measure of physical activity, was associated with improvement of the MMT-8 and reduction of CK [15].

A number of methods of assessing physical activity are available. The gold-standard measurement of energy expenditure, and therefore physical activity, is the “doubly labelled water” (DLW) method [18]. DLW is water with hydrogen and oxygen molecules replaced by traceable isotopes. Following ingestion and attainment of equilibrium within the body, serial blood or urine measurements of the concentration of the isotopes can be used to estimate the body’s metabolic rate. Although accurate, this technique is time-consuming, expensive, and not suited to measuring physical activity over prolonged continuous periods in a “real-world” setting (i.e. when the study participant is going about their daily activity).

**Fig. 1:**
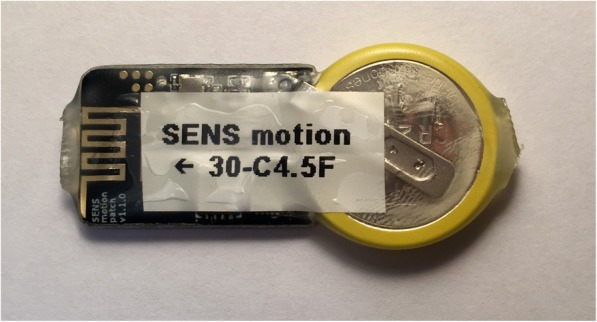
Image of internal components of an accelerometer device – thigh worn SENS Motion Plus device, which measures 20 mm × 50 mm × 3.5 mm. Reproduced with kind permission of SENS Innovation

The need for physical activity measurement in real-world settings has given rise to a number of more practical methods. One such technique is the use of accelerometer devices. Accelerometers are small, non-invasive, lightweight, portable devices that can measure acceleration in one or more geometric plane (Fig. 1). Modern “capacitive” accelerometers comprise a small micro electro-mechanical system with a proof mass attached to the end of a cantilever beam, which is surrounded by a set of fixed beams. External acceleration deflects the proof mass and generates a variation of capacitance between the fixed beams. This capacitance variation generates an electrical signal, which is then converted into a digital or analog output. This output can be used to quantify acceleration in a particular directional plane [19, 20].

Accelerometers typically measure acceleration in a single plane (uniaxial) or three orthogonal planes (triaxial). Accelerometers are capable of measuring tri-axial acceleration at high sampling rates, typically 50–100 Hz. Sampling at such a high rate over prolonged periods of time provides a temporal characterisation of physical activity, thus enabling detection of frequent (i.e. daily) changes. The acceleration data can either be analysed in its “raw” format or processed into a number of “composite” measures, such as number of steps in a time period, distance travelled or intensity of physical activity (typically categorised as sedentary, light, moderate or vigorous). Therefore, composite outputs from accelerometer-containing devices can be used to objectively summarise physical activity and identify temporal changes, for example differentiating periods of physical activity from sedentary behaviour, or identifying changes in levels of activity following an intervention. The interpretation of accelerometer-derived measurement in medical research is dependent on a number of important factors, such as body site placement (e.g. wrist, thigh, lower back), duration of use, and device used. Further, study population factors, such as disease of study interest, disease duration, presence of comorbidities, control group use, and behavioural factors such as lifestyle and living environment, also greatly influence the interpretation of collected data.

Therefore, with the need for more accurate myositis disease activity assessment and the opportunity of physical activity assessment using accelerometers, a review of studies to date on this topic will provide a useful summary of current knowledge. It will also provide an understanding of future research needs in this area.

This review aims to identify studies that have used accelerometer-derived physical activity data in studies of myositis populations, collate and compare reported physical activity data and lastly, investigate if these studies identified associations between physical activity and measures of myositis disease activity.

## Methods

A narrative review was conducted to identify original articles that have used accelerometer devices in the myositis populations/cohorts. The following databases were searched from February 2000 until February 2019: Medline via PubMed, Embase via OVID and Scopus. The following medical subject headings (MeSH) terms were used to identify appropriate studies: “myositis”, “accelerometry”, “exercise test” and “exercise”. The “myositis” MeSH term encompasses the DM, PM, and inclusion body myositis subtypes. Each identified study’s references were also examined for further appropriate studies. Studies were included if they were written in English, studied physician-confirmed human myositis cases, and measured physical activity using accelerometer-containing devices. Case reports were excluded.

The abstract of each identified study was reviewed for eligibility and excluded where appropriate. Full text review of all potentially eligible studies was subsequently carried out. Only studies that fulfilled the inclusion criteria were included in the review.

Conference abstracts were not included in the search due to the likely insufficient methodology and results details required to fully compare studies and identified relationships between accelerometer-derived physical activity and measures of myositis disease activity.

Ethical approval was not required for this study.

## Results

**Fig. 2:**
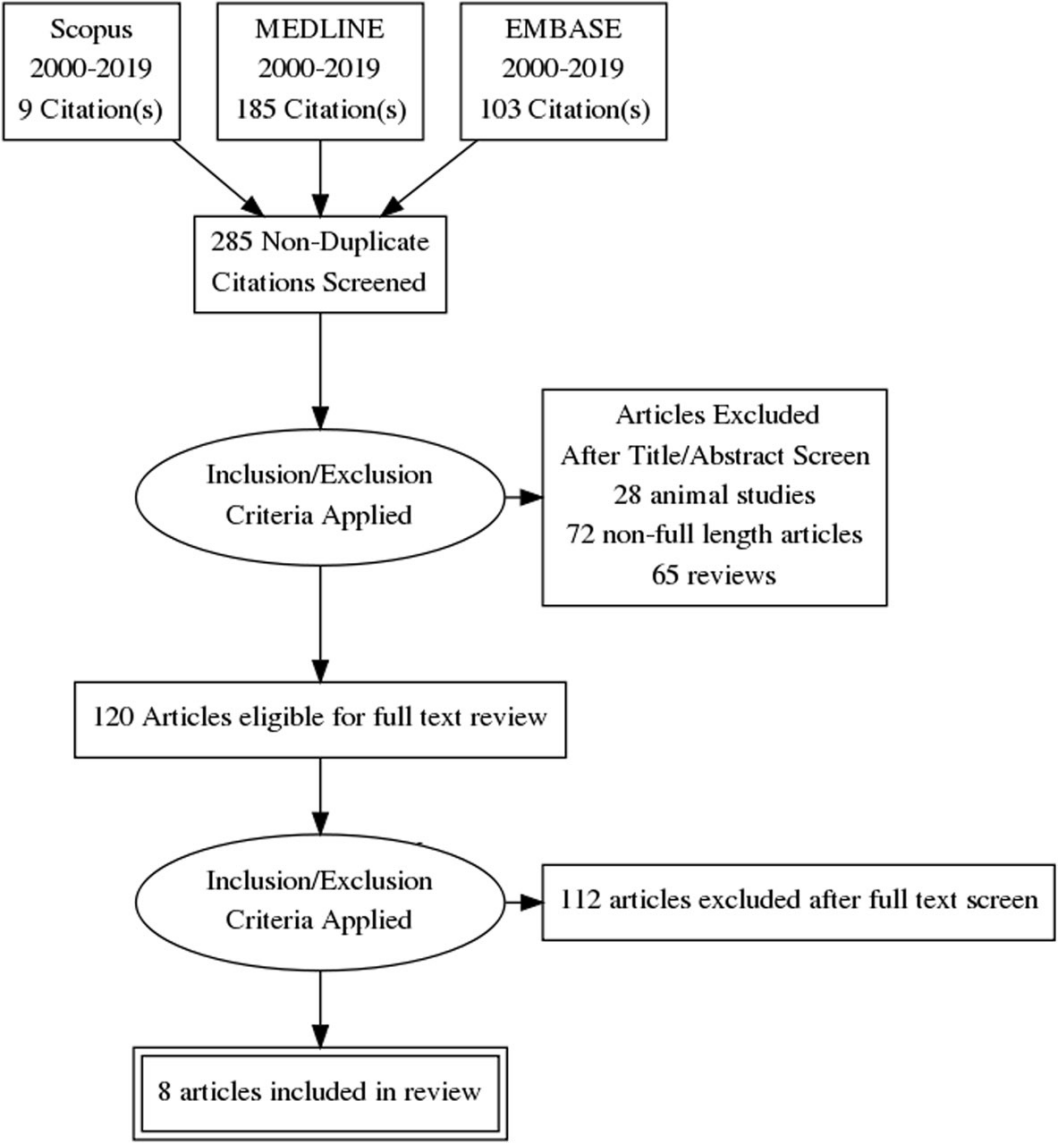
Articles identified, eligible for review and included in the narrative review

The initial search returned 297 studies. Following removal of 12 duplicates, 28 animal studies and a further 249 that did not meet the inclusion criteria, eight distinct articles, which utilised accelerometer-derived data to represent physical activity in a total of 181 myositis cases, were identified (Fig. 2), details in Tables 1 and 2. The studies varied with respect to populations investigated, devices used, site of device placement and duration of study, each of which will be considered in turn, before we compare findings and address the reported associations between physical activity and myositis disease activity.

**Table 1:** Summary of studies that met inclusion criteria – studied populations and accelerometer-specific characteristics of each study

Authors	Population	Number of participants	Duration of disease	Control group	Accelerometer device used	Duration of accelerometer data collection	Body site of accelerometer placement	Physical activity measurement	Identified associations between accelerometer-assessed physical activity and disease activity
Bachasson et al. [28]	Adult IIM cases	5 (DM = 1, IMNM = 3, ASS = 1)	Newly diagnosed		GENEActiv	14 days each month for 6 months	Wrist-worn	ENMO	Increasing ENMO followed decreasing CK and increasing SF-36 (no formal statistical test reported)
Stephens et al. [21]	JDM	15	Not reported		Actical accelerometer	7 days	Right hip	Time spent light, moderate or vigorous physical activity	Not reported
Mathiesen et al. [22]	JDM	31	Range 2–36 years		ActiGraph GT1M accelerometer	7 days	Not explicitly described^a^	Counts per minute	Not reported
Pinto et al. [23]	JDM	19	Mean 7.6 years (SD 3.2)	Healthy controls (n = 19)	ActiGraph GT3X	7 days	Elastic belt at the waistline	Time spent light, moderate or vigorous physical activity	Longer disease duration was associated with more time spent in a sedentary state (*p*-value = 0.026) Current glucocorticoid use was associated with more time spent in a moderate to vigorous physical activity state (*p*-value = < 0.001)
Pinto et al. [24]	JDM	19	Mean 7.6 years (SD 3.2)	JSLE (*n* = 20)	ActiGraph GT3X	7 days	Elastic belt at the waistline	Time spent light, moderate or vigorous physical activity	Not reported
Riisager et al. [25]	JDM	21	Median 3.4 years (range 1.4–10.3)		Sense Wear accelerometer	Two 3 day periods of data collection, 12 weeks apart	Armband on upper arm	Number of steps in 48 h period	Accelerometer-assessed physical activity improved from 16,412 to 21,079 steps per 48 h period following a 12 week exercise programme (*p*-value = 0.015)
Habers et al. [26]	JDM	26	Median 4.4 years (0.8–11.4)	Intervention (home-based exercise programme) group vs control group	Actical	7 days – carried out three or four times throughout the study	Not explicitly described^a^	Time spent in inactive, light, moderate or vigorous physical activity	No change in accelerometer-assessed physical activity between intervention (treadmill and strength exercise programme) and control groups (*p*-values = > 0.05)
Berntsen et al. [27]	JDM	45	Mean 20.8 years (SD 11.9)	Healthy controls (*n* = 45)	Actigraph GT3X	8 days	Dominant hip	Time spent in inactive, light, moderate or vigorous physical activity. Counts per minute	Shorter mean daily time spent in moderate to vigorous physical activity in inactive JDM group, compared to active JDM group (*p*-value = < 0.01)

^a^Manufacturer recommends the device to be worn on the waist at the mid-axillary line

*IIM* Idiopathic inflammatory myopathy, *JDM* Juvenile dermatomyositis, *DM* Dermatomyositis, *IMNM* Immune-mediated necrotising myopathy, *ASS* Anti-synthetase syndrome, *SD* Standard deviation, *JSLE* Juvenile systemic lupus erythematosus, *CK* Creatine kinase, *SF-36* 36 Item Short Form Survey, *MMT-8* Manual muscle testing, *ENMO* Euclidean norm minus one

**Table 2:** Reported accelerometer-derived physical activity levels in myositis cohorts

Authors		Counts per minute	Mean no. steps in 48 h	Sedentary % of day	Light % of day	Moderate % of day	Vigorous % of day	MVPA % of day
Riisager et al. [25]	Pre-training *N* = 21		16,412					
	Post-training		21,079					
Habers et al. [26] ^a^	Pre-training *N* = 26			83.0	14.0	2.8	0.1	2.9
	Post-training			80.0	15.0	4.6	0.1	4.7
Stephens et al. [21] ^b^	*N* = 15			37.7	12.3	1.4	0.6	2.2
Pinto et al. [23] ^b^	JDM cohort *N* = 19			69.4	28.0			3.7 ^c^
	Control cohort *N* = 19			66.1	29.3			4.6 ^c^
Berntsen et al. [27] ^b^	“Active” disease *N* = 16	351 ^d^		38.0	11.9			3.5 ^e^
	“Inactive” disease *N* = 29	321 ^d^		40.6	11.8			3.0 ^e^
	Control group *N* = 45	423 ^d^		39.4	11.3			4.2 ^e^
Mathiesen et al. [22] ^b^	< 18 years of age N = 19	513						
	> = 18 years of age *N* = 12	322						

^a^Accelerometer device was worn throughout 24 h periods for 7 days

^b^Accelerometer data from non-sleeping hours was analysed

^c^*P*-value > 0.05 derived from Mann-Whitney U-test

^d^*P*-value < 0.01 derived from Wilcoxon signed rank test

^e^*P*-value < 0.01 derived from t-test

*JDM* Juvenile dermatomyositis, *MVPA* Moderate to vigorous physical activity

### Populations investigated

Seven out of the eight studies used accelerometers in JDM populations [21–27] and only one, Bachasson et al. [28], reported on an adult-onset population. Both Mathiesen et al. and Berntsen et al. reported the findings from populations comprising participants both younger than and older than 18 years of age – however all study participants had experienced myositis onset aged younger than 18 years. Population sizes ranged from five to 45 study participants. Disease duration prior to study commencement varied between each study, from newly diagnosed cases to 36 years after disease onset. Bachasson et al. was the only study to report accelerometer data that was collected from the time of first treatment following diagnosis [28].

### Devices

**Fig. 3:**
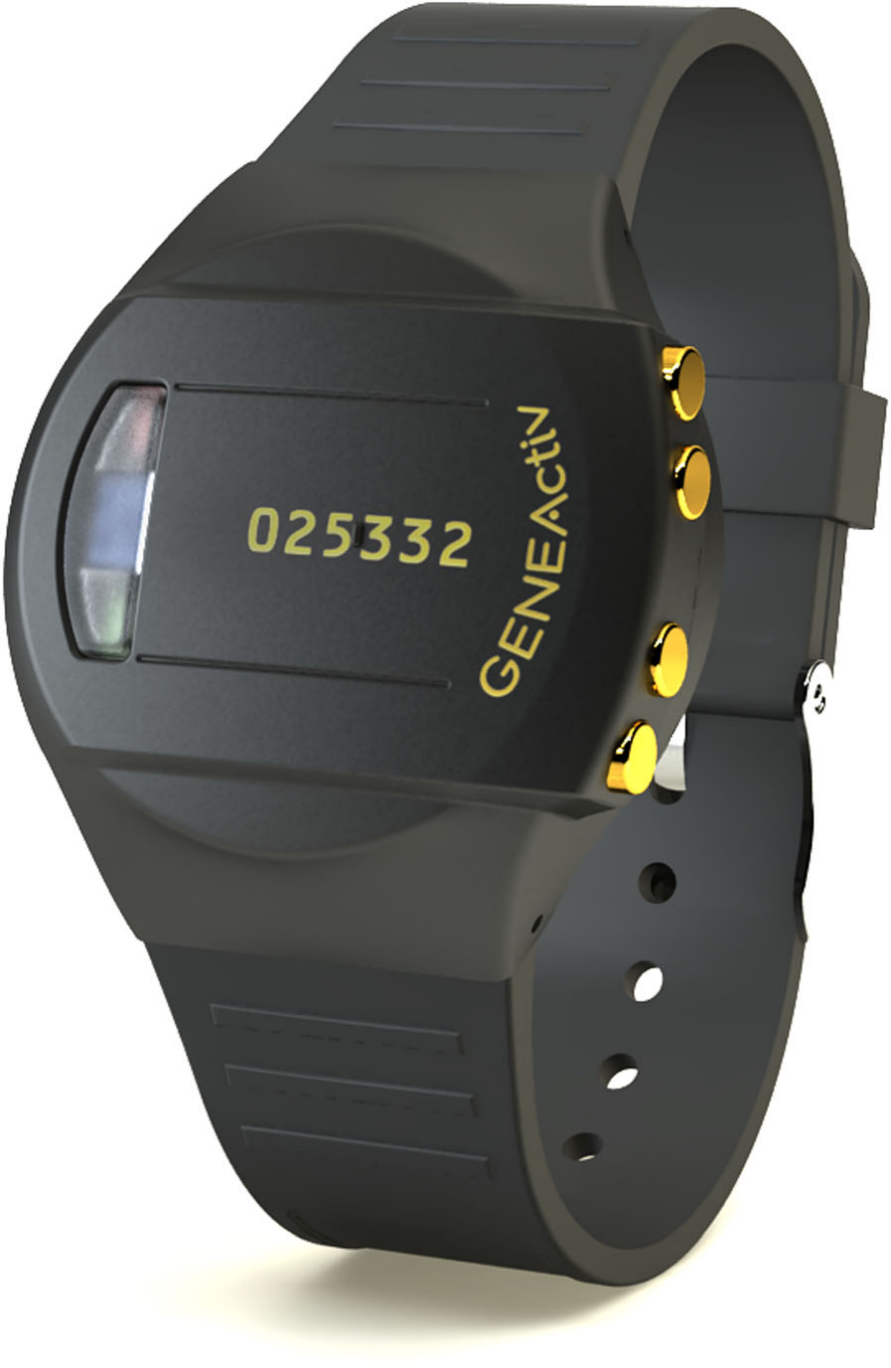
Wrist worn GENEActiv accelerometer device. Reproduced with kind permission of Activinsights

Actical, ActiGraph, Sense Wear and GENEActiv (Fig. 3) devices were used. Each accelerometer-containing device collects acceleration magnitude multiple times each second and/or provides a summarized measure of physical activity. Measures of physical activity included the “Euclidean Norm Minus One” (GENEActiv device) [28], time spent in light, moderate or vigorous states (Actical, ActiGraph GT3X devices) [21, 23, 24, 26, 27], “counts” per minute (CPM, ActiGraph GT1M device) [22, 27] and number of steps recorded in 48 h (Sense Wear device) [25].

The study by Bachasson et al. was the only one not to report a summary measure of physical activity, such as step count [28]. They reported the mean daily “vector magnitude”; the vector magnitude was calculated as the “Euclidean Norm Minus One” (ENMO). ENMO is calculated by summing the squared acceleration of each of the three accelerometer axes at each time point (i.e. Euclidean Norm) and then subtracting the gravitational component, which is 1 g (1 g  =  9.81 m/s^2^). The assumption is that increases of the mean daily vector magnitude indicates increasing levels of physical activity. Vector magnitude, being a simple mathematical operation, may preserve the relevant complexity and variation of physical activity; this contrasts to complex, composite measures such as step count which may lose important variation because in practice, these algorithms are confounded by unknown factors and developed for different populations than the one under study.

Six studies reported summary variables related to intensity of physical activity, measured in “counts”, as collected by ActiGraph GT3X and Actical devices [21–24, 26, 27]. The number of counts in a minute can be used as a proxy representation of intensity of physical activity. A single count represents an acceleration measurement exceeding a pre-specified threshold. Subsequently, each time period is assigned as corresponding to sedentary (< 100 cpm), light (> 100 and < 2295 cpm), and moderate-to-vigorous (> 2295 cpm) activity, depending on the number of counts detected in a minute. Mathiesen et al. was the only study to report CPM, without subsequently ascribing inactive, light, moderate or vigorous intensity [22].

Riisager et al. used a Sense Wear body monitoring system [25], which detects steps based on accelerometer data using a data-driven machine learning algorithm – i.e. steps are detected when the pattern of collected accelerometer data correspond to step-associated signals; however, details of the algorithm used to detect steps is not available as it is proprietary information. The number of steps per 48 h period was reported as their surrogate measurement of physical activity.

### Site of device placement

A wide variety of body sites for accelerometer placement were used in the reviewed studies, including wrist [28], upper arm [25], waist [23] and hip [21, 27]. Studies by Mathiesen et al. [22] and Habers et al. [26] did not explicitly state what site was used, however the manufacturers of the employed accelerometer devices advise them to be worn on the hip at the mid-axillary line. The site of accelerometer placement has an important impact upon study methodology, data interpretation and analysis. For example, walking speed estimation may vary between arm and thigh-worn accelerometers, as arm swing may limit accurate estimation. Studies have attempted to identify the most appropriate site of body placement in healthy states and certain disease areas [29–32], however each research question necessitates careful consideration of body site placement to ensure the provision of appropriate data. With myositis predominantly affecting proximal limb muscles and subsequently affecting gait, as described previously, it is plausible that lower limb placement would be most appropriate.

### Duration of data collection

The duration of accelerometer data collection varied between each study. Duration of accelerometer data collection periods ranged from seven to 84 days. Most studies collected accelerometer-derived data continuously throughout 7 day periods. However, studies by Riisager et al. [25] and Habers et al. [26] recorded two separate periods of accelerometer data, prior to and following 12 week exercise intervention programmes, with the aim of assessing for the effect of the intervention upon physical activity. A data collection period long enough to detect changes of disease activity is required; short 7 day periods may limit the ability to detect substantial change. The use of two separate data collection periods by Riisager et al. and Habers et al. may improve the ability to detect changes in disease activity without the need for prolonged, continuous periods.

### Accelerometer-derived physical activity levels in myositis populations

Quantifiable levels of accelerometer-derived physical activity were reported by a number of the identified studies (Table 2) and comparison across studies revealed a number of relationships.

Time Spent in MVPA was the most commonly reported accelerometer-derived physical activity measurement [21, 23, 26, 27]. Across all studies, where reported, myositis populations spent similar proportions of time in MVPA, ranging from 2.2–3.7% (prior to intervention, where applicable), thus indicating consistency, despite variations in devices employed and populations studied.

When compared to control cohorts, physical activity levels appeared to be lower in myositis cohorts; however study limitations, such as small populations, limit identification of definitive differences. Both Pinto et al. [23] and Berntsen et al. [27] demonstrated that their JDM populations spent less time in MVPA, compared to healthy controls: Pinto et al. reported that their JDM cohort spent 3.7% of each day in MVPA, compared to 4.6% in the control cohort, although this difference did not reach statistical significance (Mann-Whitney U-test *p*-value > 0.05), possibly in part due to the small (*n* = 19) cohort size. Berntsen et al. [27] demonstrated that their JDM group spent 3.5% of each day in MVPA, compared to 4.2% in the control group (t-test *p*-value < 0.01). Berntsen et al. also demonstrated a significantly lower CPM level in their JDM group, compared to the control group: 351 vs 423 CPM, respectively (Wilcoxon signed rank test *p*-value < 0.01). Mathiesen et al. [22] reported mean counts per minute from a JDM cohort – 513 in those younger than 18 years. Although they did not directly compare these findings with a healthy control population within the same study, they did compare against reference values from a study of healthy 9 and 15 year old children by Andersen et al. [33], who reported similar physical activity levels ranging 412–789 CPM. These comparisons suggested no statistically significant difference to the age and sex-matched healthy controls.

### Associations between accelerometer-derived physical activity data and myositis disease activity

Five out of the eight studies investigated associations between accelerometer-derived physical activity data and myositis disease activity variables/states. Only one of these studies (Bachasson et al. [28]) primarily aimed to investigate this association whilst the other studies reported associations whilst investigating other relationships [23, 25–27].

Bachasson et al. [28] and Riisager et al. [25] each investigated the association between accelerometer-derived physical activity and a number of myositis disease activity measurements including muscle strength via MMT-8 scores, which, as discussed previously, is a valid measurement of disease activity. Bachasson et al. reported a positive association, whereas Riisager et al. did not. Bachasson et al. found that improvement of accelerometer-derived data (ENMO), using GENEActiv devices, followed longitudinal improvements in MMT-8 scores, CK levels and Short Form Health Survey (SF-36) questionnaire scores over the first 6 months after diagnosis and treatment initiation. No formal statistical analysis was carried out due to the small study population size (*n* = 5). The ENMO ranged from 12 to 22 mili g (a unit of acceleration) at baseline and 22 to 45 mili g after 6 months of treatment. This increase, however, may not represent a meaningful change, with a study by Bakrania et al. demonstrating GENEActiv-derived mean ENMO values of 8 mili g for standing still and 65 mili g for “self-paced free living walking” [34]. Although increased ENMO was associated with stronger (higher) MMT-8 scores, the relatively high baseline values (range 105–140, maximum value = 150) precluded detection of substantial increases after 6 months of treatment (range 145–150), due to ceiling effect. The observed increase of ENMO also corresponded with reductions of CK (baseline range 1375–6366 IU/L, 6 months after treatment initiation range 50–300 IU/L), which indicates reducing disease activity, and increase in SF-36 scores (baseline range 20–40, 6 months after treatment initiation range 48–90), which indicate improving quality of life. The authors therefore concluded that associations exist between myositis disease activity and ENMO-derived physical activity.

Pinto et al. [23] investigated associations between physical activity intensity (represented by number of counts per minute recorded via Actigraph GTX accelerometers: < 100 cpm = sedentary, > 100 and < 2295 cpm = light and > 2295 cpm = moderate-to-vigorous) and a number of myositis disease activity measures including the CMAS, the CHAQ, disease activity score (DAS), manual muscle testing, CK levels, current and cumulative dose of glucocorticoids and disease duration [23]. Analysis was performed via calculation of Pearson’s correlation coefficients; no adjustment for potential confounding variables, such as age or gender, was performed. They identified that increased time spent in a sedentary state (*r* = 0.65, *p*-value = < 0.01) and shorter time in moderate to vigorous physical activity state (*r* = − 0.51, *p*-value = 0.03) was associated with longer JDM disease duration. Further, they identified that more time spent in a moderate to vigorous physical activity state was associated with current glucocorticoid use (*r* = 0.75, *p*-value = < 0.01). They reported no association between physical activity and the CHAQ (*r* = − 0.27, *p*-value = 0.27) and a number of other myositis disease activity assessment methods, including the PEDS-QL (*r* = 0.07, *p*-value = 0.78).

Riisager et al. [25] measured physical activity (via the number of steps detected in a 48 h period using an accelerometer-containing Sense Wear armband) along with the MMT-8 and the CMAS, prior to and following a 12 week exercise bike programme. Although the number of steps in a 48 h period increased following the training programme (16,412 to 21,079, p-value 0.02), no corresponding change in MMT-8 or CMAS were identified (no raw figures or statistical comparison results were supplied).

Habers et al. [26] also investigated the change in accelerometer-derived physical activity (represented by percentage of time spent in inactive, light, moderate and vigorous activity states, as measured by an Actical device over a 7 day period) in a JDM population following a 12 week treadmill and strength exercise programme [26]. In contrast to the associations identified by Riisager et al. [26], Habers et al. [26] reported no change in accelerometer-derived physical activity following the exercise intervention. This is despite improvement of the median parental disability score (0.22 vs 0.18), as part of the CHAQ, which, as discussed previously, is a valid measurement of JDM myositis disease activity [26]. Statistical comparison of the pre and post-intervention values was not reported. Therefore, although direct associations between accelerometer-derived physical activity and myositis disease activity measurements were not investigated by Habers et al. [26], the absence of improvement in physical activity despite improvement of the CHAQ indicates that an association between the two may not exist.

The study by Berntsen et al. was the only one to compare physical activity levels between those with “active” and “inactive” disease activity, according to the PRINTO criteria for clinically inactive disease [35]. Disease duration, gender distribution and disease duration were similar between the two groups. The inactive group (*n* = 29) demonstrated similar, but significantly lower, physical activity levels to the active group (*n* = 16), according to duration in MVPA (3% vs 3.5% of day, respectively) and CPM (321 vs 351, respectively, Wilcoxon signed rank test *p*-value < 0.01). Although significant, the differences between the “active” and “inactive” groups are likely not substantial enough to constitute clinically meaningful differences. Associations with disease activity measurements were not investigated for, however both CPM and MVPA duration were found to be significantly associated with maximal oxygen uptake (VO_2max_).

## Discussion

The purpose of this narrative review was to 1) identify studies that have collected accelerometer-derived physical activity data in studies of myositis populations, 2) collate and compare reported physical activity data and 3) investigate if these studies identified associations between physical activity and measures of myositis disease activity.

Firstly, we have identified that the use of accelerometer-derived physical activity data in myositis research is limited. The cause is likely multifactorial, with limited awareness of the potential benefits of accelerometer use, additional cost incurred and limited analysis expertise, each contributing. Additionally, the small number (*n* = 8) of studies that have collected such data do so under incompatible protocols, which makes direct comparison challenging and may account for some of the conflicting findings across these studies. No study included more than 45 participants, thus potentially limiting the ability to form clear conclusions. Forming a study cohort large enough for sufficient statistical power is limited by the rarity of the IIMs (incidence of 11/million person-years, prevalence of 14/100,000).

Accelerometer use in myositis is still in its infancy, and so it is useful to reflect on how such devices are furthering knowledge in other disease areas. Studies have been able to differentiate the severity of stroke by comparing morning peak of accelerometer-derived physical activity [36, 37]. In multiple sclerosis, disease-specific “count cut-points” were developed, thus allowing intensity of physical activity to be measured [38, 39]. However, in musculoskeletal disease, where there is a direct link between disease and locomotion, research has to date been less extensive but is beginning to provide important insights. For example, it has been demonstrated that accelerometer-derived data can detect improvement of physical activity following treatment initiation in patients with rheumatoid arthritis [40].

Quantification of physical activity using proportion of time spent in MVPA appeared consistent across studies of myositis populations and, where available, comparison of MVPA and CPM against healthy controls indicated lower physical activity levels. It is likely that the observed reduced MVPA and CPM are due to diminished muscle strength capability and exercise tolerance as a result of active myositis or myositis-induced muscle damage. Therefore, accelerometer-derived physical activity measurements may provide a useful method of quantification of differences of exercise tolerance between myositis and control populations, however further dedicated research in larger longitudinal cohorts will be required to fully clarify this capability.

A subset of the reviewed studies (*n* = 5 [23, 25–28]) have revealed insights into associations between accelerometer-derived physical activity data and myositis disease activity. Higher levels of physical activity were associated with lower CK (indicating diminished myositis) and improved SF-36 in an adult cohort [28] and shorter disease duration and current glucocorticoid use (mean dose 4.2 mg/day) in a juvenile population [23]. Further, the utility of accelerometer-derived physical activity data to detect changes following a 12 week exercise programme in a JDM cohort was illustrated by Riisager et al. where step count per 48 h increased [25]. This is in contrast to a study by Habers et al. which reported no detection of change in accelerometer-derived physical activity following a 12 week exercise intervention, despite changes in muscle function tests [26]. These studies’ findings also indicate that a relationship between accelerometer-derived physical activity and a number of disease activity measures (including the CHAQ, CMAS and MMT-8) may not exist, as no significant associations were identified. Detection of associations between accelerometer-derived physical activity and changes in the MMT-8 may have been limited by a ceiling effect, as demonstrated by Bachasson et al. [28]. Only one study compared accelerometer-derived physical activity between myositis cases with “active” and “inactive” disease. Interestingly, significantly lower levels of physical activity (CPM and mean daily MVPA duration) were reported in the “inactive” group. Unmeasured factors, such as degree of muscle damage, current treatment and involvement in previous exercise programmes was not reported. Therefore, unfortunately, the limited number of studies and their sample sizes preclude firm conclusions, but it remains plausible that physical activity may be a useful future surrogate measure for myositis disease activity with some early, promising observed associations.

Further research to investigate the utility of accelerometer-derived physical activity data in the IIMs and identify associations with myositis disease activity is warranted. Quantification of myositis disease activity would ideally be carried out longitudinally alongside continuous collection of accelerometer-derived physical activity data. In addition to disease activity, the IMACS Core Set Measures can quantify cumulative damage and differentiate between the two [7]. Therefore, a study to investigate the relationship between serial changes of accelerometer-derived physical activity data and the IMACS Core Set Measures may be the most appropriate approach. This approach could be complemented by additional frequent (i.e. daily) collection of disease activity proxy-measurements, such as patient reported outcome measurements; this approach has shown promise in a recent study in a population with rheumatoid arthritis [41]. None of the identified studies used the high sampling rate of accelerometers to identify changes of physical activity across short time periods, such as day-to-day. This approach has provided important insights in other disease areas, such as Parkinson’s disease [42]. Investigation into the association between short term (e.g. daily) temporal changes of physical activity in IIM cases could identify previously unrecognised variation of disease activity and potentially response to treatments in a clinical trial setting. Further, no identified study collected accelerometer data over periods longer than 6 months. Measurement of long term changes of accelerometer-derived physical activity may aid IIM disease course characterisation and identify factors predictive of relapse and remission, such as demographics, clinical features or the presence of myositis specific autoantibodies [43].

Identification of the appropriate method of collection of accelerometer data in IIM populations is required. Standardisation will improve comparison between studies and should allow replication of significant findings. Aspects of standardisation to be investigated include type of device, bodily site of placement, duration of data collection and reporting data format (i.e. raw data vs. derived physical activity measures). However, important disease manifestation differences within the IIMs must be considered, for example predominantly proximal muscle weakness in DM, compared to distal weakness in inclusion body myositis; investigation into each IIM subtype should therefore be considered, allowing focused standardisation.

The most appropriate method of processing and analysing accelerometer-derived data for the IIMs may be distinct from other musculoskeletal conditions and should be identified. For example, particular gait variations in the IIMs, as discussed previously may impact identification of steps and calculation of step count. A full description of the wide variety of algorithms for processing and analysing accelerometer-derived data is outside the scope of this review, however the implementation of “machine learning” techniques for data segmentation and detailed physical activity characterisation, which have proven fruitful in other rheumatological disease areas [44, 45], is promising.

## Conclusions

In summary, this narrative review has identified and summarised the small number of studies that have used accelerometer-derived physical activity measures in IIM populations and investigated for associations with myositis disease activity. Promisingly, a subset of these studies identified that a number of validated measures of myositis disease activity are associated with accelerometer-derived physical activity, including CK level, disease duration and glucocorticoid use. However, limited or no association was found with a number of other disease activity measures, including the CHAQ, CMAS and MMT-8. Further research into this potentially worthwhile area is warranted, with the aim of developing the most appropriate method of collection of accelerometer-derived physical activity data in IIM populations and clearly delineating relationships with disease activity measures.

## Data Availability

The datasets used and/or analysed during the current study available from the corresponding author on reasonable request.

## Abbreviations

CHAQ: Childhood Health Assessment Questionnaire
CK: Creatine kinase
CMAS: Childhood Myositis Assessment Score
DAS: Disease activity score
DLW: Doubly labelled water
DM: Dermatomyositis
ENMO: Euclidean norm minus one
FI-2: Functional Index-2
HAQ-DI: Health Assessment Questionnaire - Disability Index
IMACS: International Myositis Assessment and Clinical Studies Group
JDM: Juvenile dermatomyositis
MAP: Myositis Activity Profile
MeSH: Medical subject headings
MMT: Manual muscle testing
MVPA: Moderate to vigorous physical activity
PEDS-QL: Paediatric Quality of Life Inventory
PM: Polymyositis

## References

[1] Dobloug C, Garen T, Bitter H, Stjärne J, Stenseth G, Grøvle L (2015). Prevalence and clinical characteristics of adult polymyositis and dermatomyositis; data from a large and unselected Norwegian cohort. Ann Rheum Dis.

[2] Svensson John, Arkema Elizabeth V., Lundberg Ingrid E., Holmqvist Marie (2017). Incidence and prevalence of idiopathic inflammatory myopathies in Sweden: a nationwide population-based study. Rheumatology.

[3] Ng KP, Ramos F, Sultan SM, Isenberg DA (2009). Concomitant diseases in a cohort of patients with idiopathic myositis during long-term follow-up. Clin Rheumatol.

[4] Oldroyd A, Lilleker J, Chinoy H (2017). Idiopathic inflammatory myopathies - a guide to subtypes, diagnostic approach and treatment. Clin Med J R Coll Physicians London.

[5] Clarke AE, Bloch DA, Medsger TA, Oddis CV (1995). A longitudinal study of functional disability in a national cohort of patients with polymyositis/dermatomyositis. Arthritis Rheum.

[6] Cox FM, Titulaer MJ, Sont JK, Wintzen AR, Verschuuren JJGM, Badrising UA (2011). A 12-year follow-up in sporadic inclusion body myositis: an end stage with major disabilities. Brain.

[7] Rider LG, Werth VP, Huber AM, Alexanderson H, Rao AP, Ruperto N (2011). Measures of adult and juvenile dermatomyositis, polymyositis, and inclusion body myositis: physician and patient/parent global activity, manual muscle testing (MMT), health assessment questionnaire (HAQ)/childhood health assessment questionnaire (C-HAQ). Arthritis Care Res (Hoboken).

[8] Regardt M, Basharat P, Christopher-Stine L, Sarver C, Björn A, Lundberg IE (2015). Patients’ experience of myositis and further validation of a myositis-specific patient reported outcome measure - establishing Core domains and expanding patient input on clinical assessment in myositis. Report from OMERACT 12. J Rheumatol.

[9] Alemo Munters L, van Vollenhoven RF, Alexanderson H (2011). Patient preference assessment reveals disease aspects not covered by recommended outcomes in Polymyositis and Dermatomyositis. ISRN Rheumatol.

[10] Alexanderson H, Lundberg IE, Stenström CH. Development of the myositis activities profile--validity and reliability of a self-administered questionnaire to assess activity limitations in patients with polymyositis/dermatomyositis. J Rheumatol. 2002;29 http://www.jrheum.org/content/29/11/2386. Accessed 14 Sept 2017. 12415597

[11] WHO. Physical activity: WHO; 2017. http://www.who.int/topics/physical_activity/en/. Accessed 13 Feb 2018

[12] Chinoy Hector, Cooper Robert G. (2015). Polymyositis and dermatomyositis.

[13] Harris-Love MO, Shrader JA, Koziol D, Pahlajani N, Jain M, Smith M (2009). Distribution and severity of weakness among patients with polymyositis, dermatomyositis and juvenile dermatomyositis. Rheumatology (Oxford).

[14] Siegel KL, Kepple TM, Stanhope SJ (2007). A case study of gait compensations for hip muscle weakness in idiopathic inflammatory myopathy. Clin Biomech (Bristol, Avon).

[15] Alexanderson Helene, Regardt Malin, Ottosson Christina, Alemo Munters Li, Dastmalchi Maryam, Dani Lara, Lundberg Ingrid E. (2018). Muscle Strength and Muscle Endurance During the First Year of Treatment of Polymyositis and Dermatomyositis: A Prospective Study. The Journal of Rheumatology.

[16] Alemo Munters L, Dastmalchi M, Katz A, Esbjörnsson M, Loell I, Hanna B (2013). Improved exercise performance and increased aerobic capacity after endurance training of patients with stable polymyositis and dermatomyositis. Arthritis Res Ther.

[17] Josefson A, Romanus E, Carlsson J (1996). A functional index in myositis. J Rheumatol.

[18] Speakman JR, John R. Doubly labelled water : theory and practice: Chapman & Hall; 1997. http://www.springer.com/gb/book/9780412637803. Accessed 27 Oct 2017

[19] Kavanagh JJ, Menz HB (2008). Accelerometry: a technique for quantifying movement patterns during walking. Gait Posture.

[20] Chen KY, Bassett DR (2005). The technology of accelerometry-based activity monitors: current and future. Med Sci Sports Exerc.

[21] Bachasson D, Landon-Cardinal O, Benveniste O, Hogrel J-Y, Allenbach Y (2017). Physical activity monitoring: a promising outcome measure in idiopathic inflammatory myopathies. Neurology.

[22] Stephens SL, Tremblay MS, Faulkner G, Beyene J, Nguyen TH, Koohsari S (2016). Validity of the stage of exercise scale in children with rheumatologic conditions. J Rheumatol.

[23] Mathiesen PR, Orngreen MC, Vissing J, Andersen LB, Herlin T, Nielsen S (2013). Aerobic fitness after JDM--a long-term follow-up study. Rheumatology.

[24] Pinto AJ, Yazigi Solis M, de Sá Pinto AL, Silva CA, Maluf Elias Sallum A, Roschel H (2016). Physical (in) activity and its influence on disease-related features, physical capacity, and health-related quality of life in a cohort of chronic juvenile dermatomyositis patients. Semin Arthritis Rheum.

[25] Pinto AJ, Roschel H, Benatti FB, de Sá Pinto AL, Sallum AME, Silva CA (2016). Poor agreement of objectively measured and self-reported physical activity in juvenile dermatomyositis and juvenile systemic lupus erythematosus. Clin Rheumatol.

[26] Riisager M, Mathiesen PR, Vissing J, Preisler N, Ørngreen MC (2013). Aerobic training in persons who have recovered from juvenile dermatomyositis. Neuromuscul Disord.

[27] Habers GEA, Bos GJFJ, van Royen-Kerkhof A, Lelieveld OTHM, Armbrust W, Takken T (2016). Muscles in motion: a randomized controlled trial on the feasibility, safety and efficacy of an exercise training programme in children and adolescents with juvenile dermatomyositis. Rheumatology (Oxford).

[28] Berntsen KS, Edvardsen E, Hansen BH, Flatø B, Sjaastad I, Sanner H (2019). Cardiorespiratory fitness in long-term juvenile dermatomyositis: a controlled, cross-sectional study of active/inactive disease. Rheumatology.

[29] Cleland VJ, Schmidt MD, Salmon J, Dwyer T, Venn A (2011). Correlates of pedometer-measured and self-reported physical activity among young Australian adults. J Sci Med Sport.

[30] Boerema S, van Velsen L, Schaake L, Tönis T, Hermens H (2014). Optimal sensor placement for measuring physical activity with a 3D accelerometer. Sensors.

[31] Ladlow P, Nightingale TE, McGuigan MP, Bennett AN, Phillip R, Bilzon JLJ (2017). Impact of anatomical placement of an accelerometer on prediction of physical activity energy expenditure in lower-limb amputees. PLoS One.

[32] Urbanek JK, Harezlak J, Glynn NW, Harris T, Crainiceanu C, Zipunnikov V (2017). Stride variability measures derived from wrist- and hip-worn accelerometers. Gait Posture.

[33] Andersen LB, Harro M, Sardinha LB, Froberg K, Ekelund U, Brage S (2006). Physical activity and clustered cardiovascular risk in children: a cross-sectional study (the European youth heart study). Lancet.

[34] Bakrania K, Yates T, Rowlands AV, Esliger DW, Bunnewell S, Sanders J (2016). Intensity thresholds on raw acceleration data: Euclidean norm minus one (ENMO) and mean amplitude deviation (MAD) approaches. PLoS One.

[35] Lazarevic D, Pistorio A, Palmisani E, Miettunen P, Ravelli A, Pilkington C (2013). The PRINTO criteria for clinically inactive disease in juvenile dermatomyositis. Ann Rheum Dis.

[36] Serra MC, Balraj E, DiSanzo BL, Ivey FM, Hafer-Macko CE, Treuth MS (2017). Validating accelerometry as a measure of physical activity and energy expenditure in chronic stroke. Top Stroke Rehabil.

[37] Strømmen AM, Christensen T, Jensen K (2014). Quantitative measurement of physical activity in acute ischemic stroke and transient ischemic attack. Stroke.

[38] Sandroff BM, Motl RW, Suh Y (2012). Accelerometer output and its association with energy expenditure in persons with multiple sclerosis. J Rehabil Res Dev.

[39] Weikert M, Motl RW, Suh Y, McAuley E, Wynn D (2010). Accelerometry in persons with multiple sclerosis: measurement of physical activity or walking mobility?. J Neurol Sci.

[40] Prioreschi A, Hodkinson B, Tikly M, McVeigh JA (2014). Changes in physical activity measured by accelerometry following initiation of DMARD therapy in rheumatoid arthritis. Rheumatology (Oxford).

[41] Austin L, Sharp CA, van der Veer SN, Machin M, Humphreys J, Mellor P, et al. Providing “the bigger picture”: benefits and feasibility of integrating remote monitoring from smartphones into the electronic health record. Rheumatology. 2019. 10.1093/rheumatology/kez207. 10.1093/rheumatology/kez207 PMC7223265 31335942

[42] Fisher JM, Hammerla NY, Ploetz T, Andras P, Rochester L, Walker RW (2016). Unsupervised home monitoring of Parkinson’s disease motor symptoms using body-worn accelerometers. Parkinsonism Relat Disord.

[43] Betteridge Z, McHugh N (2016). Myositis-specific autoantibodies: an important tool to support diagnosis of myositis. J Intern Med.

[44] Kobsar D, Osis ST, Hettinga BA, Ferber R (2015). Gait biomechanics and patient-reported function as predictors of response to a hip strengthening exercise intervention in patients with knee osteoarthritis. PLoS One.

[45] Andreu-Perez J, Garcia-Gancedo L, McKinnell J, Van der Drift A, Powell A, Hamy V (2017). Developing fine-grained Actigraphies for rheumatoid arthritis patients from a single accelerometer using machine learning. Sensors.

